# Maternal use of drug substrates of placental transporters and the effect of transporter-mediated drug interactions on the risk of congenital anomalies

**DOI:** 10.1371/journal.pone.0173530

**Published:** 2017-03-13

**Authors:** Aizati N. A. Daud, Jorieke E. H. Bergman, Monika P. Oktora, Wilhelmina S. Kerstjens-Frederikse, Henk Groen, Jens H. Bos, Eelko Hak, Bob Wilffert

**Affiliations:** 1 University of Groningen, Groningen Research Institute of Pharmacy, PharmacoTherapy, -Epidemiology & -Economics, Groningen, the Netherlands; 2 Universiti Sains Malaysia, School of Pharmaceutical Sciences, Discipline of Clinical Pharmacy, Penang, Malaysia; 3 University of Groningen, University Medical Center Groningen, Department of Genetics, Groningen, the Netherlands; 4 University of Groningen, University Medical Centre Groningen, Department of Epidemiology, Groningen, the Netherlands; 5 University of Groningen, University Medical Center Groningen, Department of Clinical Pharmacy and Pharmacology, Groningen, the Netherlands; University of Cambridge, UNITED KINGDOM

## Abstract

**Background:**

A number of transporter proteins are expressed in the placenta, and they facilitate the placental transfer of drugs. The inhibition of P-glycoprotein (P-gp) was previously found to be associated with an increase in the risk of congenital anomalies caused by drug substrates of this transporter. We now explore the role of other placental transporter proteins.

**Methods:**

A population-based case-referent study was performed using cases with congenital anomalies (N = 5,131) from EUROCAT Northern Netherlands, a registry of congenital anomalies. The referent population (N = 31,055) was selected from the pregnancy IADB.nl, a pharmacy prescription database.

**Results:**

Ten placental transporters known to have comparable expression levels in the placenta to that of P-gp, were selected in this study. In total, 147 drugs were identified to be substrates, inhibitors or inducers, of these transporters. Fifty-eight of these drugs were used by at least one mother in our cases or referent population, and 28 were used in both. The highest user rate was observed for the substrates of multidrug resistance-associated protein 1, mainly folic acid (6% of cases, 8% of referents), and breast cancer resistance protein, mainly nitrofurantoin (2.3% of cases, 2.9% of referents). In contrast to P-gp, drug interactions involving substrates of these transporters did not have a significant effect on the risk of congenital anomalies.

**Conclusions:**

Some of the drugs which are substrates or inhibitors of placental transporters were commonly used during pregnancy. No significant effect of transporter inhibition was found on fetal drug exposure, possibly due to a limited number of exposures.

## Introduction

Drug use in pregnancy raises many concerns about the risk of harmful effects on the foetus while the use of these medications is inevitable to control certain medical conditions. The potential harmful effects of drugs on the foetus are dependent upon, among others, the concentration of drug that reaches the foetal circulation, a factor which is partly modulated by placental transport of drugs.

A number of transporter proteins are expressed in the placenta to facilitate the transport of biological substances to and from the foetus, including a subset of drugs [1–4]. This transport can be modulated by interactions with other drugs transported by the same transporter. These interactions may result in changes in substrate concentration in the foetal circulation without affecting the maternal blood or plasma concentration of substrate drugs [5]. The effect of drug interactions mediated by P-glycoprotein (P-gp), the most studied transporter protein, on foetal drug exposure has been described earlier [6–11]. From our previous study, the risk of specific foetal congenital anomalies was increased when the mothers used P-gp substrates in combination with other substrates or inhibitors [11].

To date, the effects of drug interactions mediated by other placental transporters were observed only in *in vitro* studies [5,12,13]. Therefore, we aimed to describe the user rates of drugs transported by placental transporters during the first trimester of pregnancy using population-based databases. The second objective was to investigate the effect of drug interactions mediated by these transporters on foetal drug exposure by assessing the changes in the risk of congenital anomalies.

## Materials and methods

### Cases sampling

Cases were selected from EUROCAT Northern Netherlands (NNL), a population-based registry for children with congenital anomalies born in the Northern provinces of the Netherlands. EUROCAT NNL registers foetuses or children with major congenital anomalies diagnosed before or after birth, and up to 10 years old, upon consent for their parents. The information available in the database includes sociodemographic characteristics of the parents and lifestyle during pregnancy. The information on drug intake was obtained from pharmacy records and then verified by a telephone interview with the mothers. Drug use was coded using the Anatomical Therapeutic Chemical (ATC) codes, and noted either as prescribed or over-the-counter (OTC).

Cases of major and minor congenital anomalies were classified according to EUROCAT Subgroup of Congenital Anomalies version 2012 [14], the International Classification of Diseases (ICD) coding system 9th revision for cases registered until 2001, and ICD 10th revision for cases registered from 2002 onwards. We included only major anomalies: anomalies of the nervous system, eye, ear, face & neck, heart, respiratory, oro-facial clefts, digestive system, urinary, genital, and limb (Table A in S1 File).

There are 6,059 cases, excluding cases with chromosomal anomalies, born between January 1, 1997 and December 31, 2013 and registered in EUROCAT NNL in March 2015. This number includes only those children whose mothers had a history of medication use at any time during pregnancy in order to match with the referent population of drug users from the prescription database. We excluded 572 cases with genetic disorders, i.e. microdeletion and monogenic disorders. To avoid selection bias in drug prescribing, we included only the first malformed child or pregnancy, which resulted in 5,131 cases.

### Referent population sampling

The referent population was selected from IADB.nl, a population-based prescription database in the Netherlands. IADB.nl holds the pharmacy data from about 600,000 people, covering several parts of the country, mostly in the Northern provinces. The data were collected from 60 participating community pharmacies, and the prescription rates of the IADB.nl population were found to be representative for the population in the Netherlands as a whole [15]. Prescriptions registered in the database were prescribed by general practitioners or specialists, which include the name of the drugs, the dispensing date, the ATC codes, dose and quantity dispensed [15].

For studies on drug use in pregnancy, Pregnancy IADB.nl was constructed based on the main IADB.nl, with linkage of prescription data of mother and child based on the coding of home address. The date of conception was determined by assuming a gestational age 273 days before the date of birth of the linked child. Twin or triplet pregnancies were excluded because the gestation period is likely to be shorter than singleton pregnancies. Details of the linkage and their validation are as reported earlier [16]. We selected children born within the same time period as the cases, whose mothers were registered with complete information on drug use. We then selected only the first registered pregnancy (N = 31,311) to avoid selection bias in the drugs prescribed, since the drug selection may be influenced by the outcome of a previous pregnancy. Since EUROCAT NNL and Pregnancy IADB.nl cover a similar geographical area, it is possible that some children were registered in both databases. We therefore excluded 256 children (0.8% out of 31,311) from the Pregnancy IADB.nl, because the birth dates of the mothers and children, and the gender of the child matched with the children in EUROCAT NNL.

### Drug exposure definitions

#### Selection of placental transporters proteins and drug substrates

Our previous study showed that P-gp inhibition was associated with an increased risk of congenital anomalies caused by drug substrates of P-gp, suggesting the importance of this transporter in foetal drug exposure [11]. Therefore, in this study, we included all other placental transporters that have a mRNA expression level in the placenta at least comparable to that of P-gp, which is 0.0255 as a ratio of the expression of peptidylprolyl isomerase (PPIA) mRNA, a housekeeping gene [17–19]. From these transporters, protein expression of BCRP, OCT3, OAT4 and OATP2B1 was detected in the first trimester placenta so far [20–22]. The protein expression of BCRP increases throughout gestation but later decreases within the third trimester, while OCT3 is moderately increased throughout gestation [21,23,24]. OAT4 and OATP2B1 were found to be expressed in the placenta of early pregnancy, although the changes in the expression throughout gestation were not much reported [25,26]. For the other transporters, we found no data regarding the protein expression in the placental layer in the first trimester of pregnancy.

The selection of drug substrates of the transporters is based on review articles that report the results from *in vitro* and *in vivo* studies [3,5,27–30]. The articles were searched in PubMed using combinations of these keywords: "placenta", "drug transporters", "drug substrate", "drug inhibitor", "Breast Cancer Resistance Protein", "ABCG2", "Multidrug resistance-associated proteins", "MRP1", "Organic anion transporters", "OAT4", "OATP2B1", "OCT3", "Monocarboxylate transporters”, "MCT1", "MCT4", "MCT8", "MCT10", "equilibrative nucleoside transporters", and "ENT1". These drugs were classified according to substrate affinity to each placental transporter, including substrate, substrate/inhibitor, inhibitor, substrate/inducer and inducer, as previously done [11].

#### User rates of drugs associated with placental transporters

For the first objective, we described the user rates of drugs associated with transporter proteins between cases and referent population. User rates were defined as the self-reported use of (in cases), or the pharmacy dispensing of (in referent population) the selected drugs, from three months before the estimated conception date through the first three months of pregnancy. We included the preconception period because the drugs may be continually used during the first trimester of pregnancy, for the referent population. The use of OTC drugs was disregarded because the Pregnancy IADB.nl does not register the use of these drugs. However, prescribing regulations for ranitidine, ibuprofen and aspirin changed in the Netherlands during the study period, as reported by the Medicines Evaluation Board of the Netherlands. Therefore, we only included the use of prescribed ranitidine, ibuprofen and aspirin among cases. Since folic acid is usually taken as an OTC medication, its use was also included only when it was prescribed.

#### Drug interactions and the risk of congenital anomalies

For the second objective, we explored the effect of drug-drug interactions involving placental transporter proteins on the risk of foetal teratogenicity. We assessed the risk of overall congenital anomalies with the use of all substrates of each placental transporter. The analyses were performed for individual transporters, as fetal drug exposure may depend upon whether the influx or efflux has changed, and the localization of the transporters. Since breast cancer resistance protein (BCRP) is known to be involved in a vectorial transport with organic anion transporting polypeptide 2B1 (OATP2B1) [1], we also determined the effect of drug interactions involving the substrates of both placental transporters.

### Statistical analysis

The user rates of the selected drugs were calculated by using the total number of cases or the population as the denominator. For the drug interaction study, binary logistic regression was used to calculate odds ratios (OR) and 95% confidence intervals for the risk of anomalies with each drug interaction pattern. Analyses were performed using PSAW Statistics, Version 22 (IBM Corporation, Armonk, NY, USA).

## Results

### Selection of placental transporters and drug substrates

**Fig 1:**
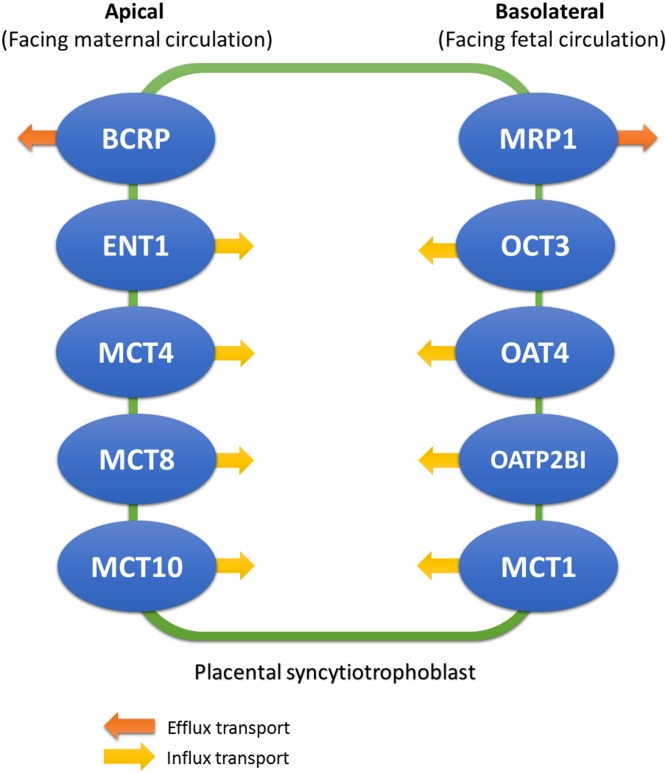
Placental transporter proteins are expressed on either side of the placenta. Five transporter proteins are expressed in the apical (maternal-facing) layer of placental cells, which include breast cancer resistance protein (BCRP), equilibrative nucleoside transporter (ENT), monocarboxylate transporter 4, 8, 10 (MCT4, MCT8, MCT10). Another five are expressed in the basolateral (fetal-facing) layer of placental cells, including multidrug resistance-associated protein (MRP), organic cation transporter 3 (OCT3), organic anion transporter 4 (OAT4), organic anion transporting polypeptide 2B1 (OATP2B1) and monocarboxylate transporter 1 (MCT1). The arrows show the direction of substrate transport through the cells.

Our study focused on placental transporters that have comparable expression levels to P-gp in the placenta, and we have identified ten eligible placental transporters: breast cancer resistance protein (BCRP) and multidrug resistance-associated protein 1 (MRP1) as efflux transporters, and organic cation transporter 3 (OCT3), equilibrative nucleoside transporter (ENT1), organic anion transporter 4 (OAT4), organic anion transporting polypeptide 2B1 (OATP2B1) and monocarboxylate transporters 1, 4, 8 and 10 (MCT1, MCT4, MCT8, MCT10) as influx transporters. The localizations of these transporters in placental tissue and the direction of substrate transport are as summarized in Fig 1, although the exact localization for some transporters is not very clear [1–4,30–36].

From the literature, 168 drugs are classified to be associated with the transport of these placental transporters, either as substrates, inducers or inhibitors. We excluded 21 of these drugs because they are either experimental or veterinary drugs, or without ATC codes. There were a total of 147 drugs selected to investigate the first objective: 53 drugs associated with BCRP transport, 45 drugs with OCT3, 29 drugs with OAT4, 28 drugs with MRP1, 24 drugs with OATP2B1, 8 drugs with ENT1, 10 drugs with MCT1, 6 drugs with MCT4, and 3 drugs with both MTC8 and MCT10 (one drug may be associated with the transport of more than one transporter). The list of all placental transporters included in this study and their respective drug substrates, inducers and inhibitors can be found in Table C in S1 File.

### User rates of drugs associated with placental transporters among cases and the referent population

Characteristics of children born to case mothers and to mothers in the referent population are shown in Table 1. The mothers in the case group were slightly older than the mothers in the referent population (30.2 ± 4.6 years and 29.5 ± 4.9 years, respectively, *p*< 0.01). The majority of cases were liveborn while all the children in the referent population were liveborn. The most common types of anomalies among the case group were heart and limb anomalies (around 25% each), followed by anomalies in the digestive system (12.3%).

**Table 1:** Characteristics of children born to case mothers and referent population mothers.

Characteristics	Cases (N = 5,131)	Referent population (N = 31,055)
Maternal age at delivery*, mean years ± s.d	30.2 ± 4.6	29.5 ± 4.9
Gender, N (%)		
*Boy*	2,864 (55.8)	16,064 (51.7)
*Girl*	2,259 (44.0)	14,991 (48.3)
*Missing data*	8 (0.2)	-
Year of birth, N (%)		
*1997–1998*	665 (13.0)	5,736 (18.5)
*1999–2000*	695 (13.5)	5,492 (17.7)
*2001–2002*	634 (12.4)	4,551 (14.7)
*2003–2004*	620 (12.1)	3,954 (12.7)
*2005–2006*	637 (12.4)	3,247 (10.5)
*2007–2008*	576 (11.2)	2,927 (9.4)
*2009–2010*	658 (12.8)	2,805 (9.0)
*2011–2012*	527 (10.3)	1,983 (6.4)
*2013*	119 (2.3)	360 (1.2)
Type of birth, N (%)		
*Live birth*	4,805 (93.6)	31,055 (100.0)
*Termination of pregnancy*	224 (4.4)	0
*Stillbirth*	65 (1.3)	0
*Miscarriage (>24 weeks)*	37 (0.7)	0
Types of anomalies^a^, N (%)		
*Heart*	1,377 (26.8)	-
*Limb*	1,228 (23.9)	-
*Digestive*	633 (12.3)	-
*Urinary*	563 (11.0)	-
*Clefts*	457 (8.9)	-
*Genital*	444 (8.7)	-
*Central nervous system*	383 (7.5)	-
*Eye*, *ear*, *face and neck*	175 (3.4)	-
*Respiratory*	91 (1.8)	-

*p-value < 0.01;

s.d: standard deviation;

^a^cases with multiple congenital anomalies are represented in more than one anomaly group

Out of 147 drugs associated with placental transporters, 58 were used by at least one mother in either the cases or the referent population, as listed in Table 2. Only 28 of the drugs were used by at least one mother in both groups. Table 3 shows the percentage of mothers who used at least one of the drugs, according to their substrate specificity to each placental transporter protein. Drugs classified to be substrates/inducers or inducers of these transporters were not found to be used by our study populations. The use of MRP1 drug substrates was more common as compared to substrates of other transporters; it was observed in 6% of the case mothers and 8% of the referent population. The highest user rate was for folic acid (prescribed), as one of the substrates of MRP1. BCRP substrates were used in 3% and 3.6% of the case and referent population, respectively. The percentage resulted largely from the use of nitrofurantoin (2.3% and 2.9% in cases and referent population, respectively). Restricting the analysis for liveborn cases only showed a slight reduction in user rates for each transporter (Tables 2 and 3).

**Table 2:** User rates of selected drugs among cases and referent population, according to substrate specificity to each placental transporter.

No	Drugs	Transporter proteins										Number of users, n (%)		
		BCRP	OCT3	OAT4	MRP1	OATP2B1	ENT1	MCT1	MCT4	MCT8	MCT10	All cases (N = 5,131)	Liveborn cases (N = 4,805)	Referent population (N = 31,055)
1	Acetazolamide			Inhibitor								0	0	1 (0.003)
2	Albendazole	Substrate										0	0	1 (0.003)
3	Amantadine		Inhibitor									0	0	1 (0.003)
4	Amitriptyline		Substrate/ Inhibitor									10 (0.2)	10 (0.2)	97 (0.3)
5	Atorvastatin					Substrate/ Inhibitor		Substrate	Inhibitor			2 (0.04)	2 (0.04)	12 (0.04)
6	Bumetanide			Inhibitor								1 (0.02)	1 (0.02)	1 (0.003)
7	Candesartan			Inhibitor								0	0	5 (0.02)
8	Captopril			Inhibitor								0	0	1 (0.003)
9	Ceftriaxone			Inhibitor								1 (0.02)	1 (0.02)	2 (0.006)
10	Cimetidine	Substrate	Substrate/ Inhibitor									2(0.04)	2(0.04)	20 (0.1)
11	Ciprofloxacin	Substrate										2 (0.04)	2 (0.04)	41 (0.1)
12	Citalopram		Substrate/ Inhibitor									17 (0.3)	14 (0.3)	102 (0.3)
13	Clonidine		Substrate/ Inhibitor									0	0	3 (0.01)
14	Cyclosporine	Inhibitor			Inhibitor	Inhibitor						1 (0.02)	1 (0.02)	4 (0.01)
15	Diltiazem		Substrate/ Inhibitor									1 (0.02)	1 (0.02)	2 (0.006)
16	Dipyridamole	Inhibitor					Inhibitor	Substrate				0	0	5 (0.02)
17	Efavirenz	Substrate/ Inhibitor			Substrate/ Inhibitor							0	0	1 (0.003)
18	Ephinephrine		Substrate									0	0	10 (0.03)
19	Erythromycin	Substrate										14 (0.3)	14 (0.3)	77 (0.2)
20	Estrone					Substrate/ Inhibitor						0	0	1 (0.003)
21	Famotidine		Inhibitor									0	0	5 (0.02)
22	Fexofenadine		Substrate/ Inhibitor			Substrate/ Inhibitor						7 (0.1)	7 (0.1)	80 (0.3)
23	Flecainide		Substrate/ Inhibitor									0	0	1 (0.003)
24	Fluvastatin					Substrate			Inhibitor			0	0	1 (0.003)
25	Folic acid				Substrate							307 (6)*	288 (6)*	2471 (8)
26	Furosemide	Substrate		Inhibitor								1 (0.02)	0	14 (0.04)
27	Gabapentin							Inhibitor				0	0	4 (0.01)
28	Gemfibrozil					Inhibitor						0	0	1 (0.003)
29	Glibenclamide	Substrate				Substrate/ Inhibitor						0	0	3 (0.01)
30	Hydrochlorothiazide	Substrate										5 (0.1)	4 (0.1)	35 (0.1)
31	Ibuprofen							Inhibitor				63 (1.2) *	59 (1.2)	850 (2.7)
32	Imipramine		Substrate/ Inhibitor									0	0	4 (0.01)
33	Indomethacin				Inhibitor							7 (0.1)	7 (0.1)	18 (0.1)
34	Ketoprofen			Substrate/ Inhibitor				Inhibitor		Inhibitor	Inhibitor	0	0	3 (0.01)
35	Lamivudine	Substrate	Substrate/ Inhibitor		Substrate/ Inhibitor							0	0	1 (0.003)
36	Lopinavir	Substrate/ Inhibitor			Substrate/ Inhibitor	Substrate/ Inhibitor						0	0	1 (0.003)
37	Losartan			Inhibitor								0	0	4 (0.01)
38	Metformin		Substrate/ Inhibitor									12 (0.2)	12 (0.2)	33 (0.1)
39	Methotrexate	Substrate		Substrate/ Inhibitor	Substrate							0	0	1 (0.003)
40	Nelfinavir	Inhibitor	Substrate/ Inhibitor			Substrate/ Inhibitor						0	0	1 (0.003)
41	Nitrofurantoin	Substrate										119 (2.3)	107 (2.2)	908 (2.9)
42	Norfloxacin	Substrate										4 (0.1)	4 (0.1)	36 (0.1)
43	Ofloxacin	Substrate										1 (0.02)		13 (0.04)
44	Omeprazole	Inhibitor										48 (0.9)	43 (0.9)	290 (0.9)
45	Pravastatin			Substrate/ Inhibitor		Substrate/ Inhibitor		Substrate				0	0	1 (0.003)
46	Progesterone		Inhibitor									72 (1.4)	67 (1.4)	295 (0.9)
47	Ranitidine		Substrate/ Inhibitor									7 (0.14) *	7 (0.1)	140 (0.5)
48	Rifampicin		Inhibitor			Inhibitor						0	0	2 (0.007)
49	Rosuvastatin	Substrate				Substrate						0	0	3 (0.01)
50	Salicylic acid (aspirin)							Substrate				3 (0.17)*	2 (0.04)	1 (0.003)
51	Simvastatin					Inhibitor			Inhibitor			3 (0.06)	3 (0.1)	19 (0.1)
52	Sulfasalazine	Substrate										8 (0.2)	7 (0.1)	25 (0.1)
53	Tacrolimus	Inhibitor										0	0	3 (0.01)
54	Tenofovir		Substrate/ Inhibitor		Substrate/ Inhibitor							0	0	1 (0.003)
55	Tetracycline			Substrate/ Inhibitor								4 (0.1)	4 (0.1)	27 (0.1)
56	Valproic acid			Substrate/ Inhibitor				Substrate	Substrate			20 (0.4)	17 (0.4)	29 (0.1)
57	Valsartan			Inhibitor								0	0	2 (0.006)
58	Verapamil		Substrate/ Inhibitor									0	0	9 (0.03)

BCRP: breast cancer resistance protein; OCT: organic cation transporter; OAT: organic anion transporter; MRP: multidrug-associated protein; OATP: organic anion transporting polypeptide; ENT: equilibrative nucleoside transporter; MCT: monocarboxylate transporter;

*prescribed

**Table 3:** User rates of drugs associated with placental transporter in the first trimester of pregnancy among cases and referent population.

Placental transporters	Substrate#, n (%)			Substrate/Inhibitor#, n (%)			Inhibitor#, n (%)		
	All cases (N = 5,131)	Liveborn cases (N = 4,805)	Referent population (N = 31,055)	All cases (N = 5,131)	Liveborn cases (N = 4,805)	Referent population (N = 31,055)	All cases (N = 5,131)	Liveborn cases (N = 4,805)	Referent population (N = 31,055)
BCRP	153 (3.0)	137 (2.9)	1,131 (3.6)	0	0	2 (0.01)	49 (1.0)	44(0.92)	302 (1.0)
OCT3	0	0	10 (0.03)	56 (1.1)	53 (1.1)	480 (1.5)	72 (1.4)	67 (1.4)	303 (1.0)
OAT4	0	0	0	24 (0.47)	21 (0.44)	60 (0.19)	3 (0.06)	2 (0.04)	28 (0.09)
MRP1	307 (6.0)	288 (6.0)	2471 (8.0)	0	0	2 (0.01)	8 (0.16)	8 (0.17)	21 (0.07)
OATP2B1	0	0	3 (0.01)	9 (0.18)	9 (0.19)	100 (0.32)	4 (0.08)	4 (0.08)	25 (0.08)
ENT1	0	0	0	0	0	0	0	0	5 (0.02)
MCT1	25 (0.55)	21 (0.44)	48 (0.15)	0	0	0	63 (1.2)	59 (1.2)	857 (2.8)
MCT4	20 (0.39)	17 (0.35)	29 (0.09)	0	0	0	5 (0.10)	5 (0.10)	32 (0.10)
MCT8 & MCT10*	0	0	0	0	0	0	0	0	3 (0.01)

BCRP: breast cancer resistance protein; OCT: organic cation transporter; OAT: organic anion transporter; MRP: multidrug-associated protein; OATP: organic anion transporting polypeptide; ENT: equilibrative nucleoside transporter; MCT: monocarboxylate transporter;

^#^same mother who used more than one drug that belongs to the same substrate classification was counted once;

*same drug substrates for both transporters

### Placental transporter-mediated drug interactions and the risk of congenital anomalies

Drug interaction analysis can only be done for MRP1, BCRP and MCT1 substrates (Table B in S1 File). There were no users of OCT3, OAT4, OATP2B1 and MCT4 substrates in combination with an inhibitor to calculate the OR. Due to limited sample size, we were not able to show any significant increase in the risk of congenital anomalies with the use of drugs transported by each transporter in combination with the inhibitors.

## Discussion

Placental transporters may potentially play a role in foetal drug transfer in view of the vast range of drugs transported by these transporters. Ten placental transporter proteins were selected based on their mRNA expression profile in the placenta, since the data on the protein expression during early pregnancy were scarce. As our focus is drug teratogenicity, the expression of these transporters during early fetal development is of major interest. Therefore, the mRNA expression profile was taken as an indicator of their protein expression during this period. For ten placental transporters included in this study, 28 drugs were reported in *in-vitro* or *ex-vivo* studies to be substrates or inhibitors, and these were used by at least one pregnant woman in both the case group and the referent population. For each placental transporter, the number of mothers who used drug substrates were generally lower than for P-gp, (the use of P-gp drug substrates was 10% in cases and 12% in the referent population) [11]. This is probably because the substrates for those transporters are much less studied and therefore much less is known about them as compared to P-gp substrates.

Knowledge about the expression of most of the placental transporters in this study is relatively new, and their contribution to drug transport in the placenta has not been completely characterized. Despite that, transporter-mediated drug interactions can potentially cause clinically relevant changes in drug pharmacokinetics and drug exposure, as previously observed for P-gp, BCRP and MRPs [5,36]. In this study, we did not find significant changes in the risk for congenital anomalies by inhibitors of the placental transporters studied, possibly due to the limited number of users in each drug interaction pattern. Furthermore, there were potential biases that might affect the risk estimates. First, we only considered the use of prescribed folic acid, because the data on OTC medications were not available in the prescription records. However, we assumed a comparable rate of non-prescribed folic acid between the case group and the referent population, which might have led to a non-differential bias on our risk estimates.

There are also several other factors that should be taken into consideration in interpreting the role of placental transporters in the foetal drug exposure. First, the same efflux and influx transporters are also present in other tissues (i.e. intestine and liver), which are important for the distribution and elimination of their substrates. Drug interactions in these tissues, apart from the placenta, may affect substrate exposure to the fetus. However, these interactions did not warrant pharmacovigilance measures so far, therefore we do not expect these interactions to have a significant impact on our results. Second, there is high inter-individual variation in the expression of these transporters due to genetic variability in the genes encoding for them [4,12]. Genotype-dependent transporter-mediated drug interactions have not yet been well studied, but the effect was already observed for fexofenadine and OATP2B1 inhibition [37], and for metformin and OCT2 inhibition [38]. Third, drug pharmacokinetics in the maternal circulation are also altered during pregnancy due to normal physiological changes [31,39], and possible drug-drug interaction involving metabolic enzymes, i.e. CYP450. However, using the software-supported medications surveillance system in the Netherlands, these interactions may have been avoided during the prescribing and dispensing process. Therefore, we assumed that, at least from 2002, the clinically significant CYP-mediated drug interactions had already been avoided [40]. Fourth, placental transporters often have broad and overlapping substrate specificities, and the net effect of drug interactions involving substrates with more than one transporter is difficult to measure.

Our study has several strengths and limitations. One of the strengths is that the information on drug use was well documented in both databases. Duration of drug use is well documented with minimal recall bias, and the inclusion of mother-children pair was done over the same period of years. Further, our study is among the first to investigate the role of transporter proteins in the placenta using a population-based study. Our knowledge of foetal transfer of drugs was previously based on *in vitro* studies using cell lines and *ex vivo* placental models, which do not take into consideration the other clinical parameters involved [35,41]. Moreover, limited methods can be used to study the role of transporters in the early stage of pregnancy, which is a crucial period for foetal development.

One limitation of our study is the potential for misclassification of drug exposure in the prescription database (Pregnancy IADB.nl) because we cannot be sure that the mothers actually took the drugs dispensed. Further, the period of exposure was an estimation calculated from the date of delivery with the assumption of 273 days of gestation. If the mothers were classified as exposed when they were not, it would lead to an underestimation of the observed risk. Another limitation is that the mRNA expression, instead of protein level, is used in the selection of placental transporters, and the assumption of drug interactions at the placenta is based on drugs prescribed and not on measured drug concentrations. The list of drug substrates reported by the literature might not be complete and the substrate classification might also depend on the concentration of the respective drug. Moreover, the substrate specificity of many transporters is broad and not very well studied for placental transporters. Confounding by indication is also impossible to address because nothing is known in our databases about the medical condition for which the drug was prescribed and used. Finally, as acknowledged earlier, despite a large number of cases and referents, due to a wide variety of exposures, we had limited power to detect statistically significant effects.

In conclusion, we classified drugs based on their substrate specificity to several placental transporter proteins, and described the use of these drugs during the first trimester of pregnancy. Using the same approach as in our previous study on P-gp, we were unable to find a significant association between the inhibition of these transporters and the risk of congenital anomalies. We did not have enough power to draw conclusions on the causality, and larger databases are needed to answer this question. However, the list of substrates for each transporter may not be complete, and therefore the effect of drug interactions may be underestimated. Nonetheless, knowledge about transporter-mediated drug interactions in the placenta is clearly important for drugs with known risk of teratogenicity. Larger-scale databases are needed in the future to further denote the role of these transporters in foetal drug transport.

## Supporting information

S1 File Table A: The codings for each type of major anomalies included in the study. Table B: Risk of overall anomalies in cases exposed to drug-drug interactions mediated by placental transporter proteins. Table C: List of placental transporter proteins included in the study and the respective drug substrates/inducers/inhibitors.
(DOCX)
