# Supplementary material for: Maternal use of drug substrates of placental transporters and the effect of transporter-mediated drug interactions on the risk of congenital anomalies
Source: PLoS One. 2017 Mar 13;12(3):e0173530. doi: 10.1371/journal.pone.0173530 (PMC5348032; doi:10.1371/journal.pone.0173530)
Supplement: S1 File — Table A: The codings for each type of major anomalies included in the study. Table B: Risk of overall anomalies in cases exposed to drug-drug interactions mediated by placental transporter proteins. Table C: List of placental transporter proteins included in the study and the respective drug substrates/inducers/inhibitors. (DOCX) [file pone.0173530.s001.docx]

**Maternal Use of Drug Substrates of Placental Transporters and the Effect of Transporter-Mediated Drug Interactions on the Risk of Congenital Anomalies**

Aizati N A Daud^1,2^*, Jorieke E H Bergman^3^, Monika P Oktora^1^, Wilhelmina S Kerstjens-Frederikse^3^, Henk Groen^4^, Jens H Bos^1^, Eelko Hak^1^, Bob Wilffert^1,5^

^1^University of Groningen, Groningen Research Institute of Pharmacy, PharmacoTherapy, -Epidemiology & -Economics, , Groningen, the Netherlands

^2^Universiti Sains Malaysia, School of Pharmaceutical Sciences, Discipline of Clinical Pharmacy, Penang, Malaysia

^3^University of Groningen, University Medical Center Groningen, Department of Genetics, Groningen, the Netherlands

^4^University of Groningen, University Medical Centre Groningen, Department of Epidemiology, Groningen, the Netherlands

^5^University of Groningen, University Medical Center Groningen, Department of Clinical Pharmacy and Pharmacology, Groningen, the Netherlands

*Corresponding author

E-mail: [aizati.daud@gmail.com](mailto:aizati.daud@gmail.com)

**Short title:** Placental transporters and transporter-mediated drug interactions

Table A: The codings for each type of major anomalies included in the study

| **Types of major anomalies** | **ICD 9** | **ICD 10** |
| --- | --- | --- |
| Nervous system | 740-742 | Q00-Q07 (not Q0782) |
| Eye, ear, face & neck | 743-744 (not 74365, 74411-74413, 7443, 74491) | Q10-Q16, Q178, Q183, Q188 (not Q101-Q103, Q105, Q135) |
| Heart | 745-746,7470-7474 (not 74550) | Q20-Q26 (not Q2111) |
| Respiratory | 748 (not 74819, 748304, 74832, 74862) | Q30-Q34 (not Q309, Q314, Q315, Q320, Q331) |
| Oro-facial clefts | 7490-7492 | Q35-Q37 |
| Digestive system, excluding diaphragmatic hernia and including hypertrophic pyloric stenosis | 750-751 (not 7500, 75012, 75024, 7506, 7510) | Q38-Q45 (not Q381, Q382, Q3850, Q401, Q4021, Q430, Q4320, Q4381, Q4382) |
| Urinary | 75261, 753 (not 753101, 75334), 756721 | Q60-Q64 (not Q610, Q633), Q794 |
| Genital | 750-7524 (not 752431, 752442), 75260, 7527-7529 | Q50-Q52, Q54-Q56 (not Q544, Q5520, Q5521) |
| Limb | 75430, 75444, 75450, 75451, 754731, 7548, 7550-7554, 755505-755507, 75551, 755522, 75553, 755552-75559, 755612-7559 | Q65-Q74 (not Q653-Q656, Q662-Q669, Q67, Q680, Q6821, Q683-Q685, Q7400) |

Table B: Risk of overall anomalies in cases exposed to drug-drug interactions mediated by placental transporter proteins

| **Drug interaction patterns** | **All cases** | | **Liveborn cases only** | |
| --- | --- | --- | --- | --- |
|  | Cases (N=5,131) | Referent population (N=31,055) | Live-born cases (N=4,805) | Referent population (N=31,055) |
| **BCRP** | | | | |
| BCRP substrates^a^  alone, n (%) | 150 (2.9) | 1101 (3.5) | 134 (2.8) | 1101 (3.5) |
| BCRP substrates^a^  + inhibitor, n (%) | 3 (0.06) | 31 (0.01) | 3 (0.06) | 31 (0.01) |
| Unadjusted OR (95% CI) | 0.71 (0.22-2.35) | | 0.8 (0.24-2.64) | |
| Adjusted OR^b^ (95% CI) | 0.69 (0.21-2.28) | | 0.78 (0.23-2.58) | |
| **MRP1** | | | | |
| MRP1substrates^a^ alone, n (%) | 306 (6.0) | 2468 (7.9) | 287 (6.0) | 2468 (7.9) |
| MRP1 substrates ^a^  + inhibitor, n (%) | 1 (0.02) | 4 (0.01) | 1 (0.02) | 4 (0.01) |
| Unadjusted OR (95% CI) | 2.02 (0.23-18.1) | | 2.15 (0.24-19.3) | |
| Adjusted OR^b^ (95% CI) | 1.96 (0.22-17.70) | | 2.04 (0.23-18.46) | |
| **MCT1** | | | | |
| MCT1 substrates ^a^ alone, n (%) | 24 (0.47) | 45 (0.14) | 20 (0.42) | 45 (0.14) |
| MCT1 substrates ^a^ + inhibitor, n (%) | 1 (0.02) | 3 (0.001) | 1 (0.02) | 3 (0.001) |
| Unadjusted OR (95% CI) | 0.63 (0.062-6.34) | | 0.75 (0.073-7.66) | |
| Adjusted OR^b^ (95% CI) | 0.61 (0.06-6.26) | | 0.74 (0.072-7.66) | |
| **BCRP-OATP2B1** | | | | |
| BCRP-OATP2B1 substrates ^a^ alone, n (%) | 150 (2.9) | 1098 (3.5) | 134 (2.8) | 1098 (3.5) |
| BCRP-OATP2B1 substrates ^a^ + inhibitor, n (%) | 3 (0.06) | 34 (0.11) | 3 (0.06) | 34 (0.11) |
| Unadjusted OR (95% CI) | 0.65 (0.2-2.13) | | 0.72 (0.22-2.39) | |
| Adjusted OR^b^ (95% CI) | 0.62 (0.19-2.05) | | 0.70 (0.21-2.33) | |

BCRP: breast cancer resistance protein; MRP: multidrug-associated protein; MCT: monocarboxylate transporter; OATP: organic anion transporting polypeptide; ^a^includes substrates and substrates/inhibitors; ^b^adjusted for maternal age and child gender

Table C: List of placental transporter proteins included in the study and the respective drug substrates/inducers/inhibitors

| **Placental transporters** | **Encoding genes** | **Localization at the placental barrier** | **Efflux or influx** | **Expression level*** | **Substrates** | **Substrates/Inhibitors** | **Inhibitors** | **Inducers** |
| --- | --- | --- | --- | --- | --- | --- | --- | --- |
| Breast cancer resistance protein, BCRP | *ABCG2* | Apical | Efflux | 0.0954 | Cimetidine, Abacavir, Lamivudine, Prazosin, Rosuvastatin, Glibenclamide, Cervivastatin, Zidovudine, Methotrexate, Furosemide, Etoposide, Daunorubicin, Doxorubicin, Epirubicin, Grepafloxacin, Erlotinib, Lapatinib, Topotecan, Irinotecan, Flavonoids, Nitrofurantoin, Ciprofloxacin, Ofloxacin, Norfloxacin, Ulifoxacin, Erythromycin, Albendazole, Pitavastatin, Hydrochlorothiazide, Triamterene, Dantrolene, Riboflavin, Adefovir, Sulfasalazine [1–5] | Lopinavir, Delavirdine, Efavirenz, Atazanavir, Imatinib, Gefitinib [6] | Dipyridamole, Saquinavir, Ritonavir, Nelfinavir, Cyclosporine, Tamoxifen, Tacrolimus, Sirolimus, Nicardipine, Nitrendipine, Nimodipine, Reserpine, Omeprazole [4,7,8] |  |
| Organic cation transporter 3, OCT3 | *SLC22A3* | Basolateral | Influx | 0.146 | Epinephrine, Histamine, Norepinephrine, Amiloride, Amphetamines  [9–12] | Cimetidine, Impiramine, Despiramine, Clonidine, Procainamide, Flecainide, Amiodarone, Verapamil, Diltiazem, Citalopram, Amitriptyline, Cisplatine, Oxaliplatine, Abacavir, Emtricitabine, Saquinavir, Tenofovir, Lamivudine, Ritonavir, Indinavir, Nelfinavir, Ranitidine, Metformin, Cocaine, Fexofenadine  [6] | Quinidine, Rifampicin, Prazosin, Phenoxybenzamine, Progesterone, Amantadine, Ketamine, Memantine, Quinine, Atropine, Diphenhydramine, Etilefrine, Famotidine, Pentamidine, Phenformin [13] |  |
| Organic anion transporter 4, OAT4 | *SLC22A11* | Basolateral | Influx | 0.106 |  | Pravastatin, Tetracycline, Zidovudine, Valproic acid, Methotrexate, Ketoprofen  [6,10] | Probenecid, Captopril, Olmesartan, Telmisartan, Candesartan, Losartan, Pratosartan, Valsartan, Ethacrynate, Furosemide, Torasemide, Bumetanide, Acetazolamide, Trichlormethiazide, Chlorothiazide, Cefadroxil, Cefazolin, Cefoperazone, Cefotaxime, Ceftriaxone, Diflunisal, Phenylbutazone, Sulfinpyrazone  [4,10,14] |  |
| Multidrug-associated resistance protein 1, MRP1 | *ABCC1* | Basolateral | Efflux | 0.0224 | Cisplatin, Methotrexate, Etoposide, Vincristine, Vinblastine, Daunorubicin, Doxorubicin, Epirubicin, Folic Acid, Grepafloxacin, Glutathione, Leukotrien  [5,12,15] | Abacavir, Emtricitabine, Tenofovir, Lamivudine, Ritonavir, Indinavir, Lopinavir, Delavirdine, Efavirenz, Nevirapine, Atazanavir [6,15] | Cyclosporine, Probenecid, Sulfinpyrazone, Indometachin  [15,16] | Saquinavir [10] |
| Organic anion transporting polypeptide 2B1, OATP2B1 | *SLCO2B1* | Basolateral | Influx | 0.0557 | Fluvastatin, Rosuvastatin, Benzylpenicillin, Bosentan, Unoprostone  [17–19] | Saquinavir, Nelfinavir, Fexofenadine, Atorvastatin, Pravastatin, Glibenclamide, Repaglinide, Lopinavir, Estrone-3-sulfate  [6] | Ritonavir, Indinavir, Rifampicin, Rifamycin, Cyclosporine, Gemfibrozil, Cerivastatin, Paclitaxel, Rosiglitazone, Simvastatin  [6] |  |
| Equilibrative nucleoside transporter, ENT1 | *SLC29A1* | Apical | Influx | 0.0656 | Adenosine, Cladribine, Clofarabine, Gemcitabine, Ribavirin, Tiazofurin [5] |  | Dipyridamole, Dilazep [11] |  |
| Monocarboxylate transporter 1, MCT1 | *SLC16A1* | Basolateral | Influx | 0.091 | Dipyridamole, Dilazep, Atorvastatin, Pravastatin, Valproic acid, Salicylic acid [12] |  | Ketoprofen, Probenecid, Ibuprofen, Gabapentin [20,21] |  |
| Monocarboxylate transporter 4, MCT4 | *SLC16A3* | Apical | Influx | 4.99 | Valproic acid [12] |  | Atorvastatin, Fluvastatin, Cerivastatin, Simvastatin, Lovastatin [20–22] |  |
| Monocarboxylate transporter 8, MCT8 | *SLC16A2* | Apical | Influx | 0.0442 |  |  | Probenecid, Ketoprofen, Despiramine [20,23] |  |
| Monocarboxylate transporter 10, MCT10 | *SLC16A10* | Apical | Influx | 0.0203 |  |  | Probenecid, Ketoprofen, Despiramine [20,23] |  |

*ratio of transporter protein mRNA to PPIA (peptidylprolyl isomerase A) mRNA

**References**

1. Hutson JR, Koren G, Matthews SG. Placental P-glycoprotein and breast cancer resistance protein: influence of polymorphisms on fetal drug exposure and physiology. Placenta. Elsevier Ltd; 2010;31:351–7.

2. Gedeon C, Koren G. Designing pregnancy centered medications: drugs which do not cross the human placenta. Placenta. 2006;27:861–8.

3. Hahnova-Cygalova L, Ceckova M, Staud F. Fetoprotective activity of breast cancer resistance protein (BCRP, ABCG2): expression and function throughout pregnancy. Drug Metab. Rev. 2011;43:53–68.

4. Myllynen P, Immonen E, Kummu M, Vähäkangas K. Developmental expression of drug metabolizing enzymes and transporter proteins in human placenta and fetal tissues. Expert Opin. Drug Metab. Toxicol. 2009;5:1483–99.

5. Klaassen CD, Aleksunes LM. Xenobiotic, bile acid, and cholesterol transporters: function and regulation. Pharmacol. Rev. 2010;62:1–96.

6. Staud F, Cerveny L, Ceckova M. Pharmacotherapy in pregnancy; effect of ABC and SLC transporters on drug transport across the placenta and fetal drug exposure. J. Drug Target. 2012;20:736–63.

7. Weiss J, Dormann SG, Martin-facklam M, Kerpen CJ. Inhibition of P-Glycoprotein by Newer Antidepressants. J. Pharmacol. Exp. Ther. 2003;305:197–204.

8. Mao Q. BCRP/ABCG2 in the placenta: expression, function and regulation. Pharm. Res. 2008;25:1244–55.

9. Ho RH, Kim RB. Transporters and drug therapy: Implications for drug disposition and disease. Clin. Pharmacol. Ther. 2005;78:260–77.

10. Vähäkangas K, Myllynen P. Drug transporters in the human blood-placental barrier. Br. J. Pharmacol. 2009;158:665–78.

11. Unadkat JD, Dahlin A, Vijay S. Placental drug transporters. Curr. Drug Metab. 2004;5:125–31.

12. Syme MR, Paxton JW, Keelan JA. Drug Transfer and Metabolism by the Human Placenta. Clin. Pharmacokinet. 2004;43:487–514.

13. Nies AT, Koepsell H, Damme K, Schwab M. Organic cation transporters (OCTs, MATEs), in vitro and in vivo evidence for the importance in drug therapy. Drug Transp. Handb. Exp. Pharmacol. ISSN 0171-2004. 2011. p. 105–67.

14. Cha SH, Sekine T, Kusuhara H, Yu E, Kim JY, Kim DK, et al. Molecular cloning and characterization of multispecific organic anion transporter 4 expressed in the placenta. J. Biol. Chem. 2000;275:4507–12.

15. Endres CJ, Hsiao P, Chung FS, Unadkat JD. The role of transporters in drug interactions. Eur. J. Pharm. Sci. 2006;27:501–17.

16. Gulati A, Gerk PM. Role of placental ATP-binding cassette (ABC) transporters in antiretroviral therapy during pregnancy. J. Pharm. Sci. 2009;98:2317–35.

17. Obaidat A, Roth M, Hagenbuch B. The expression and function of organic anion transporting polypeptides in normal tissues and in cancer. Annu. Rev. Pharmacol. Toxicol. 2012;52:135–51.

18. Roth M, Obaidat A, Hagenbuch B. OATPs, OATs and OCTs: The organic anion and cation transporters of the SLCO and SLC22A gene superfamilies. Br. J. Pharmacol. 2012;165:1260–87.

19. Hua WJ, Hua WX, Fang HJ. The role of OATP1B1 and BCRP in pharmacokinetics and DDI of novel statins. Cardiovasc. Ther. 2012;30:e234-41.

20. Morris ME, Felmlee MA. Overview of the proton-coupled MCT (SLC16A) family of transporters: characterization, function and role in the transport of the drug of abuse gamma-hydroxybutyric acid. AAPS J. 2008;10:311–21.

21. Meredith D, Christian HC. The SLC16 monocaboxylate transporter family. Xenobiotica. 2008;38:1072–106.

22. Kobayashi M, Otsuka Y, Itagaki S, Hirano T, Iseki K. Inhibitory effects of statins on human monocarboxylate transporter 4. Int. J. Pharm. 2006;317:19–25.

23. Loubière LS, Vasilopoulou E, Glazier JD, Taylor PM, Franklyn JA, Kilby MD, et al. Expression and function of thyroid hormone transporters in the microvillous plasma membrane of human term placental syncytiotrophoblast. Endocrinology. 2012;153:6126–35.
